# Work and Worry

**Published:** 1874-04

**Authors:** 


### WORK AND WORRY.

The mind often kills the body. This is more frequently seen in men who have heavy responsibilities in connection with a nervous temperament—such as merchants, railway managers, superintendents of extensive manufactories. Then, there is another class of cases in the occupation of men who have a multitude of perplexing and intricate details to attend to; those who are called on constantly to suppress their emotions, almost habitually so, as is often the case with women; also those which involve great and sometimes violent vicissitudes; these are they who oftenest suffer from a broken down nervous system—making life a burden, and, in many cases, so unendurable that relief is sought in suicide, whose fate would often be regarded with sincerest commiseration if but a thousandth part of the sorrow was known under which the poor heart suffered.

Many a person reads with indifference, if not with a kind of contempt, the details of a suicide, or dismisses it from the mind most carelessly, as if to say, "Oh, he was crazy; it's a good riddance; better dead than alive." In almost every case of deliberate suicide it may be taken for granted that life was crushed out by some sorrow or wounding, which broke the poor heart that could no longer stand up against its grief.

There are three remedies: the first, and greatest, and best are the consolations of religion; getting out of the one ruinous rut of thought; embarking in some new enterprise, which compels outdoor activities, which shall ensure pleasureable results.

